# Nonerosive gastroesophageal reflux disease and mild degree of esophagitis: Comparison of symptoms endoscopic, manometric and pH-metric patterns

**DOI:** 10.1186/1477-7819-10-84

**Published:** 2012-05-16

**Authors:** Michele Grande, Pierpaolo Sileri, Grazia Maria Attinà, Elisabetta De Luca, Paolo Ciano, Carolina Ilaria Ciangola, Federica Cadeddu

**Affiliations:** 1 Department of Surgery, University Hospital Tor Vergata, Viale Oxford 81, I-00133, Rome, Italy

**Keywords:** Antireflux surgery, Gastroesophageal reflux disease, Nonerosive reflux disease, Erosive esophagitis, 24-hour pH-metry

## Abstract

**Background:**

Our aim in the present study was to compare patients presenting with gastroesophageal reflux disease in the presence or absence of mild-grade esophagitis (grade I or II according to the Savary-Miller classification).

**Methods:**

Between 2005 and 2007, 215 patients with gastroesophageal reflux disease (67 with reflux associated with grade I or II esophagitis and 148 without esophagitis) were evaluated at the Department of Surgery, University Hospital Tor Vergata, Rome, and were included in the present study. The evaluations consisted of clinical interviews, endoscopy of the high digestive tract, esophageal manometry and pH monitoring.

**Results:**

There was no significant difference between the two groups with regard to age, sex or symptoms. The incidence of heartburn associated with noncardiac chest pain was greater in the esophagitis group than in the dysphagia group. The incidence of hiatal hernia was similar in both groups. Although the motor pattern was similar in both groups, the length of the abdominal esophagus was greater in patients without esophagitis (1.6 cm vs 1.1 cm; *P* < 0.05). The reflux pattern was nearly identical in both groups.

**Conclusions:**

Gastroesophageal reflux without esophagitis must be regarded not as a milder form of the disease but as part of a single disease. Furthermore, these patients often demonstrate lower rates of symptom improvement after antireflux treatment in comparison with patients with erosive esophagitis. Therefore, further trials to assess the treatment algorithm for these patients are warranted.

## Background

Gastroesophageal reflux disease (GERD) represents an important medical problem in Western countries: about 20% of the population in Western countries complain of experiencing typical symptoms of this disease (heartburn and acid regurgitation). Furthermore, the incidence is probably underestimated, because many patients have extraesophageal symptoms such as asthma, cough, hoarseness and chest pain [1,2].

GERD is associated with a variety of lesions, including esophageal erosion, ulceration, stricture and Barrett’s esophagus. Reflux-related symptoms and lesions do not necessarily coexist, however, given that about 30% to 70% of patients who complain of typical symptoms have no signs of esophagitis based on endoscopy [1]. Therefore, nonerosive reflux disease (NERD) and erosive esophagitis (EE) represent the most common clinical features of GERD. The increasing use of 24-hour pH-metry allowed us to select patients with or without an increase in the acidification time of the esophagus.

Researchers who have evaluated differences between NERD and EE patients have found that patients with NERD have the following morphologic and functional patterns: lower esophageal sphincter (LES) pressure, minimal esophageal body motility abnormalities, lower esophageal acid exposure profile, low prevalence of hiatal hernia (HH) and minimal nighttime esophageal exposure. Moreover, NERD patients present with a lower incidence of acid reflux events than do patients with reflux esophagitis and Barrett’s esophagus.

It has been suggested that NERD is a disease in itself, different from EE and Barrett’s esophagus. Actually, it can be hard to diagnose NERD patients, either in either clinical practice or clinical research. NERD patients may present with symptoms as severe as those with EE, and the impairment in quality of life may be similar to that of GERD patients with or without endoscopically diagnosed esophagitis.

In the literature, investigators in different trials have compared patients with NERD with those with GERD, but without taking into account the degree of esophagitis. We that this represents a significant bias and could affect the data obtained from pH-metric and manometric tests.

Our aim in the present study was to compare the characteristics of reflux episodes in patients with NERD and EE, considering only those with hyperemia, edema, friability, pallor or erosions of the esophageal mucosa (grade I or II esophagitis according to the Savary-Miller classification).

## Methods

Between 2005 and January 2007, 215 patients with GERD symptoms were evaluated at the Department of Surgery, Tor Vergata University Hospital, Rome, and were included in this prospective, nonrandomized study. The study was approved by the Institutional Committee of the Tor Vergata University of Rome.

All patients underwent a complete evaluation, including esophagogastroduodenoscopy (EGDS), esophageal manometry with a six-channel computerized perfusion tube, and 24-hour esophageal pH monitoring using antimony monocrystalline electrodes with or without a separate skin reference electrode. In the presence of respiratory symptoms, antimony with two electrodes at 10 cm was adopted.

The 215 patients had neither undergone previous abdominal surgery nor used any kind of drugs influencing esophageal motility at the time of the clinical evaluation. Proton pump inhibitor (PPI) or H2 inhibitor therapy was interrupted at least 1 week before pH monitoring and esophageal manometry.

All patients with endoscopic findings of GERD were analyzed, but only patients with Savary-Miller grade I or II esophagitis were included. The following were the criteria for inclusion in the study:

1. Patients with nonerosive esophagitis symptoms: mucosal changes such as erythema, edema, friability or pallor

2. Patients with EE: erosions in the esophageal mucosa in addition to the changes mentioned in item 1

3. Absence of endoscopic evidence of GERD, but with positive pH-metry

Pathologic reflux was assessed on the basis of the pH-monitoring pattern, even in the absence of features of esophagitis upon EGDS.

In addition to demographics, the presence of heartburn, dysphagia, noncardiac chest pain (NCCP) and respiratory symptoms possibly linked to reflux were recorded. In every patient who complained of asthmalike symptoms, retrosternal chest pain and pharyngeal constriction, pneumatological or otolaryngological evaluations were performed. HH was assumed to be present if the stomach protruded more than 2 cm through the esophageal hiatus.

Manometric studies were performed to evaluate the LES for amplitude, length and capacity of relaxation upon swallowing. The features and morphology of the swallowing complexes were analyzed together with the propagation of peristaltic waves in the body of the esophagus.

Intraesophageal pH readings were recorded using a Digitrapper pH 400 (Medtronic Functional Diagnostic A/S, Skovlunde, Denmark). An antimony pH catheter (Medtronic Inc, Minneapolis, MN, USA) was inserted transnasally with the patient under local anesthesia and placed 5 cm distal to the gastric cardia, and pH readings were recorded for 24 hours.

The analysis of the pH monitoring included the following parameters:

1. Total number of reflux episodes

2. Total number of pathological episodes of reflux (pH < 4 for more than 5 minutes)

3. Percentage of reflux time compared with total monitoring time (total reflux time)

4. Percentage of reflux time compared with the time while the patient was in the upright position (reflux time in erect position)5. Percentage of reflux time compared with the time while the patient was lying down (reflux time in supine position)

5. Johnson and DeMeester composite scoring system score (based on the above-mentioned parameters) [2]

The following are the normal values of the above-mentioned parameters:

1. Total reflux time up to 3.2%

2. Reflux time in erect position up to 8.2%

3. Reflux time in supine position up to 3%

4. Johnson and DeMeester score up to 14.7 (>6 or 7 up to 10) [2]

Quality of life was assessed using the SF-36 health survey questionnaire for all patients.

### Statistical analysis

All statistical elaborations were obtained by using statistical software for Windows XP (Statgraphics, version 6). Results are expressed as values and standard deviation (means ± SD).

Nonparametric tests were used to analyze the results, taking into account the nature of the variables. The following tests were applied: Student’s *t*-test for numerical data and a *χ*^2^ test with Yates’s correction for qualitative data. In all tests, 0.05 or 5% was established as the level for rejecting the hypothesis.

## Results

Between 2005 and January 2007, 258 consecutive patients with GERD symptoms were evaluated at the Department of Surgery, Tor Vergata University Hospital, Rome. Of the 258 patients, 43 (16.7%) with Savary-Miller grade III or IV esophagitis were excluded from our study. Therefore, 215 patients were evaluated and included in this prospective, nonrandomized trial.

The baseline characteristics of the two groups are shown in Table 1. Sixty-seven patients (31.2%) reported reflux associated with Savary-Miller grade I or II esophagitis (RE patients), and 148 patients (68.8%) showed no evidence of esophagitis (RE-free patients).

**Table 1:** **Demographic characteristics of the 215 patients according to the presence or absence of reflux esophagitis**^**a**^

**Demographic variables**	**RE patients,*n*(%)**	**RE-free patients,*n*(%)**	**Total patients,*n*(%)**
Number of patients	67 (31.2)	148 (68.8)	215 (100)
Men	23 (34.3)	60 (40.5)	83 (38.6)
Women	44 (65.7)	88 (59.5)	132 (61.4)
Age range, years (median)	24 to 74 (48.87)	17 to 73 (50.10)	17 to 74 (49.72)
Total	67 (100.0)	148 (100.0)	215 (100.0)

^a^RE, reflux esophagitis.

There were no statistically significant differences between the two groups regarding presence of HH (*χ*^2^ = 0.259; *P* > 0.05), LES pressure values (*χ*^2^ = 67.50; *P* > 0.05) or total reflux time (*χ*^2^ = 55.50; *P* > 0.05). Heartburn occurred in 38.8% of RE patients and 61.5% of the RE-free group, and the difference was statistically significant (Fisher’s exact test; *P* < 0.001). A significant association between heartburn and NCCP was recorded in 38.8% of RE patients and 9.4% of the RE-free group (*χ*^2^ = 24.811; *P* = 0.0001).

Retrosternal pain was more evident in RE-free than in RE patients (23.0% vs 17.9%, *χ*^2^ = 0.260; *P* > 0.05), but the difference was not significant. Dysphagia was detected only in the RE group (4.5%, *χ*^2^ = 3.860; *P* = 0.049), and extraesophageal symptoms were noted only in the RE-free group (6.1%, *χ*^2^ = 2.871; *P* > 0.05) (Table 2).

**Table 2:** **Clinical characteristics of the 215 patients according to the presence or absence of reflux esophagitis**^**a**^

**Symptoms**	**RE patients,*n*(%)**	**RE-free patients,*n*(%)**	**Total patients,*n*(%)**	***P*values**
Heartburn	26 (38.8)	91 (61.5)	117 (51.6)	0.001
Chest pain	12 (17.9)	34 (23)	46 (21.4)	NS
NCCP heartburn	26 (38.8)	14 (9.4)	40 (18.6)	0.0001
Dysphagia	3 (4.5)	0 (0)	3 (1.4)	NS
Extraesophageal symptoms	0 (0)	9 (6.1)	9 (4.2)	NS
Total	67	148	215	

^a^*NCCP*, noncardiac chest pain; *NS*, not statistically significant.

The results of manometric studies of the esophagus were substantially similar in the two groups of patients. The only statistically relevant difference was the length of the abdominal esophagus (*χ*^2^ = 14.24; *P* = 0.05) (Table 3).

**Table 3:** **Manometric pattern of the 215 patients according to the presence or absence of reflux esophagitis**^**a**^

**Measured parameters**	**RE patients (mean values)**	**RE-free patients (mean values)**	***P*values**
LES pressure (normal value = 14.3 to 34.5 mmHg)	15.3 mmHg	16.8 mmHg	NS
Length of abdominal LES	1.1 cm	1.6 cm	0.05
Amplitude pressure waves (normal value = 64 to 154)	74.7 mmHg	73.8 mmHg	NS
Duration pressure waves (normal value = 2.9 to 5.1 seconds)	3.2 seconds	3.1 seconds	NS
Motor alteration of esophageal body	71.9%	67.9%	NS

^a^RE, reflux esophagitis; LES, lower esophageal sphincter; NS, not statistically significant.

Prolonged esophageal pH monitoring, carried out in all patients, showed no significant differences between the two groups regarding total reflux time or reflux time in either the erect or supine position (*χ*^2^ = 65.59; *P* > 0.05). There was no significant difference in Johnson and DeMeester scores between RE patients and RE-free patients (F-ratio = 0.86; *P* > 0.05) (Table 4).

**Table 4:** **pH-metric pattern of the 215 patients according to the presence or absence of reflux esophagitis**^**a**^

**Measured parameters**	**RE patients (mean values)**	**RE-free patients (mean values)**	***P*values**
Total reflux number	140.6	140.1	NS
Reflux number >5 minutes	3.8	2.7	NS
pH time <4 minutes	166.4	109.5	NS
Reflux in supine and erect	69.6%	76.9%	NS
Johnson and DeMeester score	32.8	45.5	NS

^a^RE, reflux esophagitis; NS, not statistically significant.

## Discussion

A significant number of patients with classical esophageal reflux symptoms do not show endoscopic evidence of esophagitis. This group was regarded as having an attenuated form of gastroesophageal reflux (GER).

Kasapidis *et al.*[3] defined pathologic GER patients as only those with suggestive symptoms confirmed by pH monitoring. In accord with Nasi *et al.*[4], we do not agree with that definition, which does not take into account the significant group of symptomatic patients with esophagitis and normal pH-metry. Moreover, the failure of pH-metry to identify pathologic reflux in patients with esophagitis suggests that factors other than acidity, such as pepsin and bile salts, are involved in the pathogenesis of GERD.

Patients without endoscopic or pH-metric evidence of GER have been defined as having functional heartburn [3]. This may be due to hypersensitivity to acidic or other nonacidic factors, which could fire up their heartburn. On the other hand, increased sensibility to acid of esophageal chemoreceptors was found in patients without RE, even if they had a sensitivity to these factors lower than that of patients with EE [4].

On the basis of symptoms, the differential diagnosis between GERD with RE and NERD is really challenging. In our experience, patients with NERD complained of extraesophageal symptoms and retrosternal pain more often than RE patients and had a lower incidence of dysphagia. Nonetheless, these differences were not statistically significant.

In our series, in contrast to results reported by other authors, the impact of heartburn was significantly higher in patients without esophagitis than in those with it (*P* < 0.001). Moreover, the presence of heartburn with pain was more frequent in patients with esophagitis than in those without it (*P* < 0.0001).

In agreement with several other researchers, we found that the incidence of symptoms in GERD patients with versus without esophagitis is similar [5,6]. Therefore, we cannot assume the presence of reflux disease with or without esophagitis based solely on symptoms.

EGDS in the present study detected HH in 144 patients overall (67%), comprising 47 patients (70%) in the esophagitis group and 97 patients (65.5%) in the nonesophagitis arm, without significant differences between the two groups. On the contrary, other authors have found a higher incidence of HH in patients with esophagitis [4,7]. Actually, HH is considered a risk factor because it reduces esophageal clearance [4,8-10]. Mittal *et al.*, who based their findings on esophageal scintigraphy to assess esophageal clearing time, observed that clearing time was shorter in patients with HH [9]. DeMeester *et al.* found an association between the presence of HH and severe acidic exposure of the esophageal mucosa detected by pH-metry [11].

Although total reflux time is considered the key factor in diagnosing GERD patients, in agreement with other authors, we found no difference among patients with versus those without esophagitis regarding total reflux time and Johnson and DeMeester score.

Likewise, Nasi *et al.*[4] believed that decreased resistance of the esophageal mucosa and the presence of harmful agents in the reflux (pepsin and/or bile salts), which are poorly assessed by esophageal pH monitoring, may contribute to GERD. Similarly, Pujol *et al.* found that there was no statistically significant difference in the number of episodes of reflux, time of reflux and Johnson and DeMeester score in patients with versus those without esophagitis [12].

In a pH-metric data analysis, Quigley *et al.* reported more than 75% of RE patients and about 50% to 65% of non-RE patients presented with alterations of esophageal acidification time [13]. In our experience, the presence of multiple episodes of pathological GER was found in 53.9% of patients with RE and in 52.2% of patients without RE (*P* > 0.05).

Clinically high-grade esophagitis is often encountered in association with motility dysfunction of the lower esophagus and normal LES pressure values. In our experience, similar to that described by Almeida *et al.*[14], statistical analysis of esophageal motility parameters showed no significant difference between the two groups, with the exception of the length of the intraabdominal esophagus, which was longer in the group without RE (1.6 cm vs 1.1 cm; *P* < 0.05).

Regarding the manometric pattern, in contrast to other authors who found motor impairments of the esophageal body in RE patients [4,15], we found no difference between the two groups in terms of LES pressure and abdominal and total extension of the sphincter. On the contrary, Wu *et al.* found a higher prevalence of esophageal dysmotility in non-RE patient [4,16]. Actually, patient selection is a key factor. In our study, patients with Savary-Miller grade III or IV esophagitis were not considered, in contrast to studies by other investigators who did not differentiate patients according to esophagitis grade.

Fass *et al.*[17] and Schindlbeck [18] found that in the emergency room, patient biopsies of the esophageal mucosa during EGDS could be another useful diagnostic tool. These authors reported a higher rate of inflammatory cells (for example, neutrophilic and eosinophilic granulocytes) as well as greater epithelial hyperplasia or mucosal vessel dilatation in histological specimens of both RE patients without esophagitis and non-RE patients with altered pH tests than in controls or non-RE patients with normal pH-metry [17,18].

We found no difference in quality of life between the two groups, although we did not use specific questionnaires to evaluate the quality of life in the first part of our clinical experience. Previously, patients with endoscopy-negative reflux disease were considered to have milder disease. This concept is incorrect, given that the impact on quality of life is similar in GERD patients with or without esophagitis and is related to symptoms in both cases [17-20].

In patients who present with alarm symptoms, such as dysphagia, weight loss, anorexia and anemia, EGDS should be regarded as the first-line diagnostic approach. In contrast, patients who present with typical symptoms of GERD but without alarm symptoms will most likely be treated empirically, without the patient’s or the physician’s knowing whether there is an esophageal mucosal injury [21]. Only 25% of the patients without esophagitis who initially achieved complete symptom resolution while taking PPIs will remain symptom-free after 6 months without any antireflux therapy [22]. These findings suggest that most patients without esophagitis will require long-term therapy, regardless of the treatment that initially induces symptom remission.

Therefore, we do not agree with empirical PPI therapy, especially in young patients. We suggest performing continuous 24-hour pH monitoring in all patients to exclude or confirm the presence of duodenogastroesophageal reflux. Actually, the absence of pathological reflux during pH-metric monitoring could be useful by excluding the presence of acid reflux but cannot be used to detect episodes of alkaline refluxes, which can be investigated only with spectrophotometry of biliar pigments.

## Conclusions

In conclusion, we believe that NERD does not represent a different form of GERD, but that it should be considered an aspect of this condition with unique diagnostic and therapeutic features. Therapy must be tailored to each patient according to clinical and instrumental findings, especially in those patients who show good compliance and acceptable improvement in symptoms with “on-demand” or intermittent drug administration. Finally, surgery should be considered among the therapeutic options for well-selected patients with persistent reflux-associated symptoms and no endoscopic evidence of esophagitis, but with pathological pH values.

## Competing interests

The authors declare that they have no competing interests.

## Authors’ contributions

FC, MG manuscript preparation and critical review. EDL, GMA literature review and manuscript preparation. PC, CIC data collection and literature review. PS critical review. All authors read and approved the final manuscript.

## References

[B1] Armstrong D A critical assessment of the current status of non-erosive reflux disease Digestion 2008 78 Suppl 1 46 54 18832840 10.1159/000151255

[B2] Johnson LF DeMeester TR Twenty-four-hour pH monitoring of the distal esophagus: a quantitative measure of gastroesophageal reflux Am J Gastroenterol 1974 62 325 332 4432845

[B3] Kasapidis P Xynos E Mantides A Chrysos E Demonakou M Nikolopoulos N Vassilakis JS Differences in manometry and 24-h ambulatory pH-metry between patients with and without endoscopic or histological esophagitis in gastroesophageal reflux disease Am J Gastroenterol 1993 88 1893 1899 8237938

[B4] Nasi A Filho JP Zilberstein B Cecconello I Gama-Rodrigues JJ Pinotti HW Gastroesophageal reflux disease: clinical, endoscopic, and intraluminal esophageal pH monitoring evaluation Dis Esophagus 2001 14 41 49 10.1111/j.1442-2050.2001.00130.x 11422305

[B5] Holtmann G Reflux disease: the disorder of the third millennium Eur J Gastroenterol Hepatol 2001 13 Suppl 1 S5 S11 11430509

[B6] Fass R Fennerty MB Vakil N Nonerosive reflux disease current concepts and dilemmas Am J Gastroenterol 2001 96 303 314 11232668 10.1111/j.1572-0241.2001.03511.x

[B7] Pace F Santalucia F Bianchi Porro G Natural history of gastro-esophageal reflux disease without oesophagitis Gut 1991 32 845 848 10.1136/gut.32.8.845 1885063 PMC1378949

[B8] Jenkinson LR Norris TL Watson A Influence of hiatal hernia on oesophageal function Gut 1987 28 A1377

[B9] Mittal RK Lange RC McCallum RW Identification and mechanism of delayed esophageal acid clearance in subjects with hiatus hernia Gastroenterology 1987 92 130 135 3781181 10.1016/0016-5085(87)90849-3

[B10] Sloan S Kahrilas PJ Impairment of esophageal emptying with hiatal hernia Gastroenterology 1991 100 596 605 1993483 10.1016/0016-5085(91)80003-r

[B11] DeMeester TR Lafontaine E Joelsson BE Skinner DB Ryan JW O’Sullivan GC Brunsden BS Johnson LF Relationship of a hiatal hernia to the function of the body of the esophagus and the gastroesophageal junction J Thorac Cardiovasc Surg 1981 82 547 558 7278346

[B12] Pujol A Grande L Ros E Pera C Utility of inpatient 24-hour intraesophageal pH monitoring in diagnosis of gastroesophageal reflux Dig Dis Sci 1988 33 1134 1140 10.1007/BF01535790 3409799

[B13] Quigley EM DiBaise JK Non-erosive reflux disease: the real problem in gastro-oesophageal reflux disease Dig Liver Dis 2001 33 523 527 10.1016/S1590-8658(01)80100-6 11816537

[B14] Almeida SM Estudo da motilidade esofagiana na doença do refluxo gastroesofagiano 1995 Tese (Mestrado), Faculdade de Medicina do Centro de Ciĕncias da Saúde, Universidade Federal do Rio de Janeiro, Rio de Janeiro 145

[B15] Kasapidis P Xynos E Mantides A Chrysos E Demonakou M Nikolopoulos N Vassilakis JS Differences in manometry and 24-h ambulatory pH-metry between patients with and without endoscopic or histological esophagitis in gastroesophageal reflux disease Am J Gastroenterol 1993 88 1893 1899 8237938

[B16] Wu JC Cheung CM Wong VW Sung JJ Distinct clinical characteristics between patients with nonerosive reflux disease and those with reflux esophagitis Clin Gastroenterol Hepatol 2007 5 690 695 10.1016/j.cgh.2007.02.023 17481961

[B17] Fass R Symptom assessment tools for gastroesophageal reflux disease (GERD) treatment J Clin Gastroenterol 2007 41 437 444 10.1097/MCG.0b013e31802e849f 17450022

[B18] Schindlbeck N 24-hour pH-metry of the esophagus [in German] MMW Fortschr Med 2003 145 51 52 14655515

[B19] Fass R Fennerty MB Vakil N Non-erosive reflux disease (NERD): current concepts and dilemmas Am J Gastroenterol 2001 96 303 314 11232668 10.1111/j.1572-0241.2001.03511.x

[B20] Savarino E Zentilin P Tutuian R Pohl D Casa DD Frazzoni M Cestari R Savarino V The role of nonacid reflux in NERD: lessons learned from impedance-pH monitoring in 150 patients off therapy Am J Gastroenterol 2008 103 2685 2693 10.1111/j.1572-0241.2008.02119.x 18775017

[B21] Fass R Nonerosive reflux disease MedGenMed 2001 3 1 S13 S18 [http://www.medscape.com/viewarticle/407966]

[B22] Carlsson R Dent J Watts R Riley S Sheikh R Hatlebakk J Haug K de Groot G van Oudvorst A Dalväg A Junghard O Wiklund I Gastro-oesophageal reflux disease in primary care: an international study of different treatment strategies with omeprazole. International GORD Study Group Eur J Gastroenterol Hepatol 1998 10 119 124 10.1097/00042737-199802000-00004 9581986

